# Approximal Caries Detection by DIFOTI: *In Vitro* Comparison of Diagnostic Accuracy/Efficacy with Film and Digital Radiography

**DOI:** 10.1155/2012/326401

**Published:** 2012-11-04

**Authors:** Á. Ástvaldsdóttir, K. Åhlund, W. P. Holbrook, B. de Verdier, S. Tranæus

**Affiliations:** ^1^Division of Cariology, Department of Dental Medicine, Karolinska Institutet, Huddinge, Sweden; ^2^Faculty of Odontology, University of Iceland, Reykjavík, Iceland; ^3^The Swedish Council on Technology Assessment in Health Care, Stockholm, Sweden

## Abstract

The aim of the present study was to compare the diagnostic accuracy/efficacy of digital imaging fiber-optic transillumination (DIFOTI) with film and digital radiography, in detection of approximal caries lesions. One hundred and twelve approximal surfaces were scored for caries, using DIFOTI images film and digital radiographs. All three sets of images were examined twice by 8 observers, with a minimal interval of one week between examinations. Validation of histological sections served as a reference standard. Reproducibility, based on intra- and interobserver agreement, was similar for all three methods. At diagnostic threshold D1 (enamel and dentin caries), DIFOTI showed significantly higher sensitivity, but differences in specificity between methods were nonsignificant. Diagnostic accuracy in the form of area under the receiver operating characteristic curve (AUC) was significantly higher for DIFOTI. At diagnostic threshold D3 (dentin caries), the differences in sensitivity and AUC among methods were nonsignificant, but DIFOTI showed significantly lower specificity. Compared with the radiographs, DIFOTI showed closer agreement, expressed as weighted kappa values, with the reference standard. The results show that under *in vitro* conditions, the diagnostic accuracy of DIFOTI in detecting early approximal enamel lesions is greater than that of film and digital radiography, while the potential for detecting lesions in dentin is similar for all three methods.

## 1. Introduction

The overall decline in caries prevalence and improved understanding of the pathology of the caries process [1–4] have led to a change in treatment approach. The importance of early detection and preventive intervention before the development of irreversible damage is now generally accepted [5]. However, for such a treatment approach to function in general dental practice, the dentist needs access to methods which allow not only detection of early lesions but also monitoring of the effect of interventions, that is, to observe progression, arrest, or regression of such lesions over time. There is therefore a need for more reliable and accurate detection methods than traditional visual inspection and tactile examination.

Radiography is the most common method used by clinicians to complement visual examination. The method has shown quite high sensitivity for detection of dentin caries on approximal surfaces, but is of limited value for detecting early lesions in enamel [6, 7]. Though the method is widely used in clinical practice, exposure of patients to ionizing radiation is a matter of concern. In the current context of low caries prevalence and slow progression of new lesions, it is suggested that radiographs are no longer routinely required for all patients: adequate selection criteria should be applied to determine when radiographs are indicated [8, 9]. This approach, however, precludes frequent monitoring of early lesions and response to preventive measures. 

To meet the need for more accurate and noninvasive detection methods, attempts have been made to develop instruments which will reliably detect early lesions. One such instrument is digital imaging fiber optic transillumination (DIFOTI), a refinement of its predecessor, fiber-optic transillumination (FOTI), based on transillumination of teeth with intense fiber-optic light. The optical properties of a caries lesion are different from those of the surrounding sound dental tissues, and FOTI amplifies the change in scattering and absorption of light photons in the carious tissue thereby making the caries lesion appear as a dark shadow. The method has been applied as an aid to visual examination but yields qualitative information that is nonreproducible and needs subjective interpretation. The major advantage of the method is that it is noninvasive and can therefore be as frequently used as needed. 

The DIFOTI system was designed to overcome the limitations of FOTI by providing digital image capture. Such images can be stored in digitized form and compared with previously acquired images. 

The method has been applied in a few *in vitro* studies, with somewhat contradictory results. Schneiderman et al. (1997) [10] reported that compared to conventional film radiography, DIFOTI had greater sensitivity for detection of approximal, occlusal, and smooth-surface caries lesions. However, Young and Featherstone [11] reported that DIFOTI was more sensitive than film radiography to initial surface changes, but failed to yield accurate quantitative information. 

To date, the only clinical study on diagnostic accuracy of DIFOTI concluded that the method could increase sensitivity when used in conjunction with digital or film radiography [12]. Further studies are warranted to validate the method. 

The purpose of diagnostic accuracy/efficacy studies is to determine how closely the results of the method being tested agree with a reference standard, under controlled conditions. The selected reference standard should be the best available method to confirm the true condition. In *in vitro* caries detection studies, this is achieved by histopathology of sections of the teeth.

The aim of this *in vitro* study was therefore to compare the diagnostic accuracy/efficacy of DIFOTI and conventional digital and film radiography in detection of approximal caries at two different diagnostic thresholds, using histology and microradiography as standard references.

## 2. Material and Methods

### 2.1. Sample Teeth

As the biological material comprising the study sample could not be traced to an individual donor, the regional Ethics Committee in Stockholm, Sweden, determined that the study was not subject to the law of ethical approval (2006/3:4). 

The material comprised 56 premolar teeth, extracted on orthodontic indications and stored in thymol saturated saline. The approximal surfaces of the selected teeth presented a range of conditions, from sound to noncavitated and cavitated caries lesions. There were no visually detectable caries lesions on other surfaces.

The teeth were rinsed in 10% sodium hypochlorite solution for 20 min, followed by rinsing in distilled water for 20 min. The teeth were then arranged in groups of 4 and mounted in plastic blocks, simulating the anatomic position in the dental arch. The blocks were then used to produce DIFOTI images film and digital radiographs. The teeth were kept moist and refrigerated between examinations.

### 2.2. Observers

Eight observers (ÁÁ, HD, JG, MS, LEL, PN, SEH, and ST) participated in the study. They examined each set of images twice, with a minimal interval of one week between examinations, and scored on a scale of 0–4. All examiners were experienced in the use of radiographs for clinical caries detection and quantification but only three had a research background within the field of caries detection (ÁÁ, LEL, and ST). Six observers had no previous experience of DIFOTI (HD, JG, MS, LEL, PN, and SEH). 

### 2.3. DIFOTI Images

All DIFOTI images were captured by a trained operator (ÁÁ) under standardized darkroom conditions. The DIFOTI instrument (Electro-Optical Sciences Inc, NY) was used as recommended by the manufacturer. Images were captured from three different sites: buccal, lingual, and occlusal, using both the occlusal and the approximal handpieces. Microsoft PowerPoint 2002 was then used to arrange the images so that all images for each surface appeared simultaneously. The images were viewed on a 15-inch Hewlett Packard (L1520) monitor. 

Before the DIFOTI examination, all observers underwent a 15-minute training session to become familiar with the technique. The observation sessions were then conducted in the same manner as described later for the digital radiographs. Findings were scored on a scale of 0 to 4 score 0 = no shadow, score 1 = shadow restricted to outer half of enamel, score 2 = shadow reaching inner half of enamel, score 3 = shadow reaching outer half of dentin, and score 4 = shadow reaching inner half of dentin.

### 2.4. Radiography

In order to ensure the quality of the radiographs, a pilot study had been undertaken to determine optimal exposure time, type of soft tissue equivalent, and projection geometry. Following the pilot study, a special holder used in the present study was constructed. The holder arranged the components in the following order: the cone was placed at a distance of approximately 3 cm from the teeth and 5 cm from the film or the sensor. To simulate soft tissue, a 22 mm Plexiglas was positioned directly in front of the cone.

The observations were undertaken in a darkroom, without any additional light source. A 15-inch Hewlett Packard (L1520) monitor was used for viewing the digital radiographs and DIFOTI images. Brightness and contrast were standardized. The observers were instructed to adjust their sitting position so that the images being viewed were at eye level, at a distance of approximately 50 cm. They were instructed not to change position while viewing the images. The radiographs were evaluated according to a scale of 0–4: score 0 = no visible radiolucency, score 1 = radiolucency visible in the outer half of the enamel, score 2 = radiolucency visible in the inner half of the enamel, score 3 = radiolucency visible in the outer half of the dentine, and score 4 = radiolucency visible in the inner half of the dentine.

#### 2.4.1. Film Radiography

The holder described above was used for standardization of projection geometry. The radiographs were taken using Planmeca intraoral radiographic equipment (Planmeca, Helsinki, Finland) and Kodak Ektaspeed plus films, with settings of 70 kV and 7 mA and an exposure time of 0.25 s. 

The film radiographs were placed on a table with a light source, in a darkroom with no additional light sources, and examined using Mathsson binoculars with 2-fold magnification.

#### 2.4.2. Digital Radiography

The digital radiographs were captured using the holder described above for film radiography. Focus intraoral radiographic equipment, involving a Sigma direct digital sensor and Cliniview software (Instrumentarium, Tuusula, Finland), was used. The settings were 60 kV and 7 mA, with an exposure time of 0.20 s. The images were manipulated in order to standardize brightness and contrast, using ImageJ software (ImageJ, U.S. National Institutes of Health, Bethesda, MD, USA). The images were viewed using Microsoft PowerPoint 2002 under standardized conditions, as described above. 

### 2.5. Histological Validation

After all examinations were completed, the teeth were removed from the blocks and divided buccolingually. All approximal surfaces were then sectioned, perpendicular to the enamel and the occlusal surfaces, into approximately 300 *μ*m thick sections at preselected sites, using a Buehler IsoMet low speed precision saw (Buehler, Illinois, USA). 

For microradiography, the sections were then exposed to Ni-filtered Cu K*α* radiation at 20 kV and 20 mA with an exposure time of 2 h, using Kodak high speed holographic film SO 253. After standardised development of the films, three operators (ANA, ÁÁ, and ST) examined both the microradiograms and the tooth sections independently under a stereomicroscope, at a magnification factor of 16. Lesion depths were scored on a scale from 0 to 4: 0 = no demineralization, 1 = demineralization extending to outer half of the enamel, 2 = demineralization extending to inner half of the enamel, 3 = demineralization extending to outer half of the dentin, and 4 = demineralization extending to the inner half of the dentin. This procedure for histological validation has been used at the Institute of Odontology, Karolinska Institutet, Stockholm, Sweden, for 20 years. One of the observers (ST) has 10 years' experience of the technique, and the other two observers (ANA and ÁÁ) underwent a training session prior to the histological validation.

On completion of validation, consensus was reached for histological and microradiogram observations, respectively, by adopting the most common score of all three observers for each surface. If all three disagreed, the score of the experienced examiner (ST) was used. When comparing histological and microradiogram observations, the highest score for each surface was used as a reference standard (RS).

### 2.6. Statistical Analysis

The evaluation of the reference standard was carried out by calculating weighted kappa (*κ*_*w*_) agreement among the three observers. Interexaminer agreement was calculated using *κ*_*w*_ for each pair of observers for each method. A mean *κ*_*w*_ value for each method was calculated and minimum and maximum values specified. Intraexaminer reliability was calculated in the same way, using *κ*_*w*_. Agreement between approximal caries scores and the reference standard was also calculated by *κ*_*w*_. The *κ*_*w*_ values were interpreted according to Landis and Koch [13].

Two cut-off levels, based on histology, were used for calculations of sensitivity, specificity, and AUC: area under the receiver operating characteristic curve (ROC). Cut-off 1 represents the D1 threshold: enamel and dentin caries. Cut-off 2 represents the D3 threshold: dentin caries. To compare diagnostic performance between methods, sensitivity, specificity, and AUC values were compared pairwise using nonparametric statistics (Wilcoxon). The level of statistical significance was set at *P* < 0.05. Analyse-it (Analyse-it Software, Ltd., Leeds, UK) and Statistica (StatSoft Scandinavia AB, Sweden) were used for statistical analyses. 

## 3. Results

The material comprised 112 approximal surfaces, of which 15 were irreparably damaged during preparation of the samples for histological validation. To assess the effect of this loss of material on the results, the most common score of all examiners and all methods was used. The surfaces were scored as follows: 10 as sound, 2 as enamel caries lesions and 3 as dentin caries lesions. Due to the high proportion of sound surfaces in the damaged material it is assumed that loss of these observations would not have influenced the results, as all methods showed acceptable specificity at both thresholds.

**Figure 1:**
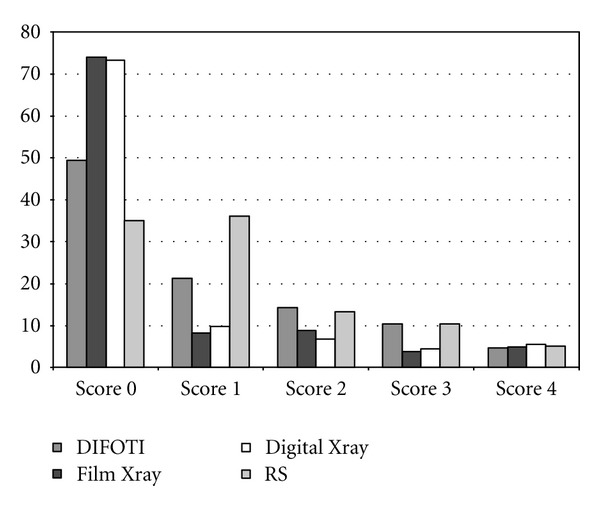
The mean percentage of each detection score for each method, compared to the distribution of the reference standard (RS).

The material was scored on a scale of 0–4. The reference standard comprised 35% score 0, 36% score 1, 14% score 2, 10% score 3, and 5% score 4 (Figure 1). The *κ*_*w*_ values for the histological validation were 0.89, 0.89, and 0.93 for each pair of examiners. The *κ*_*w*_ values for the microradiographic validation were 0.86, 0.87, and 0.91. The *κ*_*w*_ agreement between histopathology and microradiography was 0.82. 

**Figure 2:**
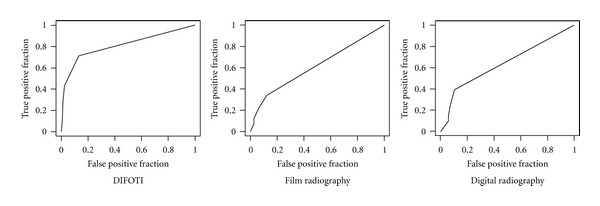
ROC curves for the three methods based on results from 8 observers at the diagnostic threshold D1 (enamel and dentin caries).

**Figure 3:**
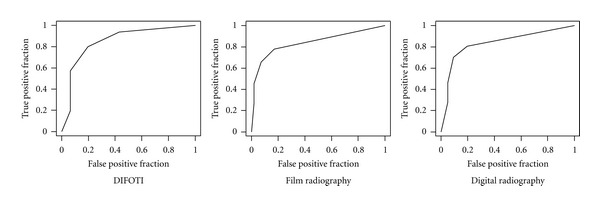
ROC curves for the three methods based on results from 8 observers at the diagnostic threshold D3 (dentin caries).

**Figure 4:**
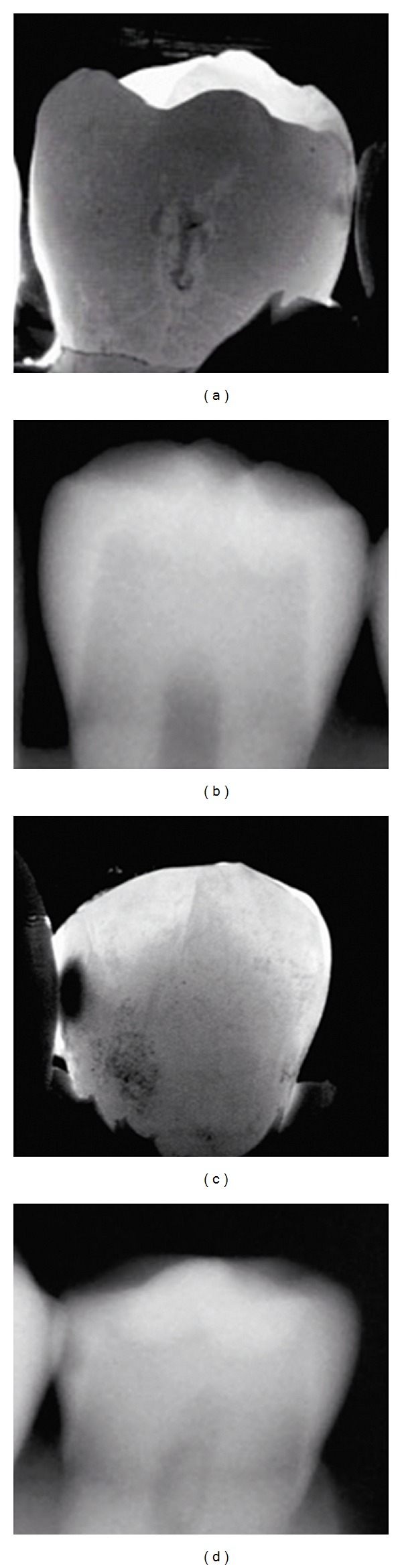
Two sets of images (a) DIFOTI image and (b) digital radiograph of lesion with reference standard score 1. (c) DIFOTI image and (d) digital radiograph of lesion with reference standard score 3.

Figure 1 shows a comparison of the mean numbers for each detection score for each method and the distribution of the true score (RS).  Figure 2 presents ROC curves for all methods at diagnostic threshold D1. In Figure 3 the ROC curves at diagnostic threshold D3 are presented. The reproducibility of each method in the form of mean intra- and interexaminer agreement, based on the eight observers participating in the study, is shown in Table 1. Sensitivity, specificity, and AUC for all examiners, for all methods, are presented in Table 2.  Table 3 presents a comparison of the methods by Wilcoxon Matched Pairs test. In Table 4, agreement between caries diagnosis and the reference standard is presented as *κ*_*w*_ values.  Figure 4 shows three sets of images, DIFOTI and digital radiography, of surfaces with score 1, 2, and 3. 

## 4. Discussion

Thorough validation is an essential step in the development of new diagnostic methods and instruments for caries detection. Initially, technical efficacy should be confirmed. Diagnostic accuracy and efficacy in identifying lesions at different diagnostic thresholds should then be determined before clinical application. 

The efficacy of a diagnostic method indicates how well the method works under controlled conditions. Under such conditions efficacy should be high, because it can be assumed that in the clinical setting, lower effectiveness will be achieved. To date there are no published studies validating the diagnostic accuracy and efficacy of DIFOTI *in vitro* at different diagnostic thresholds. Hence the present* in vitro* study, testing diagnostic accuracy in terms of sensitivity, specificity, and AUC, was undertaken as an important step in validating this instrument. 

The DIFOTI method was compared to methods more familiar to most clinicians, film and digital radiographs. When viewing radiographs, examiner performance is determined by skill and experience, viewing conditions, and the material being examined [14]. In the present study, viewing conditions were therefore standardized, and the material was selected in order to reflect the currently low prevalence of dentin caries observed in the population [2, 3]. The eight dentists who participated as observers in the study did, however, have different backgrounds and experience, and this probably influenced their performance [15, 16]. Although a 15-minute training session on the DIFOTI method was undertaken, it may be assumed that lack of familiarity with the method in combination with their experience and background may have influenced their interpretation of DIFOTI images. This probably explains in part the great variance in interexaminer agreement for all methods, while intraexaminer agreement was in general much better. With respect to reproducibility of the three methods, however, it can be concluded that no pronounced differences emerged.

When testing diagnostic accuracy and efficacy of a new method it is important to differentiate between the main and surrogate diagnostic endpoints. A main diagnostic endpoint influences treatment decisions, thereby differentiating between nonoperative treatment decisions and operative interventions. In an *in vitro* study, such an endpoint can not be obtained. Treatment decisions are not only based on lesion depth and surface integrity, but also on lesion activity and diagnosis of the patient caries activity which is not available for *in vitro* material. The two endpoints tested in this study were therefore endpoints based on lesion depth [17] and stand for the ability of the method to detect all caries lesions, even the early enamel lesion, D1, and the ability of the method to detect the more advanced form of the disease, lesion extending to dentin, D3. This should therefore be further tested under clinical conditions with better defined endpoints, thereby testing the instruments diagnostic thinking efficacy as well as therapeutic efficacy [18]. 

Detection of initial caries lesions is of high clinical relevance, as the presence of such lesions implies increased caries activity and the need for additional, noninvasive intervention intended to arrest or reverse lesion progression. In this context, the DIFOTI method allows noninvasive capture of images which can be used as a reference between appointments and thereby used to monitor changes in lesion depth.

At diagnostic threshold D1, the DIFOTI method showed significantly better sensitivity than the other methods, but similar specificity (Table 3). Thus compared to radiography, the method seems to be able to identify lesions at an earlier stage, without increasing the number of sound surfaces incorrectly identified as carious. The diagnostic accuracy of the DIFOTI method was further supported by significantly greater area under the ROC curve than the radiographs (Figure 2). These results are in accordance with earlier studies reporting low sensitivity of radiographs in detecting initial lesions [19]. The DIFOTI method has also been shown to be more sensitive than radiographs to early changes in enamel [11]. The results of the present study therefore suggest that at the D1 threshold DIFOTI images provide more accurate information than radiographs.

At the D3 level, the differences in performance between DIFOTI and radiography were less pronounced (Figure 3). The differences in sensitivity were nonsignificant, but both types of radiograph showed better specificity than DIFOTI; that is, the number of lesions incorrectly identified as dentin lesions was higher for DIFOTI than for radiography. In this context, however, it is of interest to note that as shown in Table 2, six out of the eight examiners achieved good specificity with DIFOTI. With respect to area under the ROC curve, differences between methods were non-significant, suggesting similar diagnostic accuracy for all three methods at the D3 threshold.

As shown in Table 4, the DIFOTI method showed best overall agreement with the reference standard: DIFOTI observations by six out of eight examiners showed good to very good agreement. In contrast, for film and digital radiography observations, good agreement was achieved by only two of the eight examiners. Thus the DIFOTI method showed better correlation with actual lesion depth than the other methods. These results are in contrast to those of Young and Featherstone [11], reporting that DIFOTI was inferior to radiographs in judging lesion depth. In that study, small artificial lesions were used to test the diagnostic accuracy of the method in contrast to our study where natural caries with great variance in lesion depth was examined, which might explain the difference to some extent. The instrument might be able to identify great change in lesion depth although small changes in the mineral content of the enamel lesion are not detectable. 

The results of the present study therefore suggest that DIFOTI records lesion depth more accurately than radiography. It should, however, be borne in mind that the material involved a high proportion of enamel caries (Figure 1) which can favor the DIFOTI method.

In conclusion, the results suggest that within the limitations of the study, the diagnostic accuracy/efficacy of DIFOTI is superior to radiography at diagnostic threshold D1 and comparable to radiography at diagnostic threshold D3. Lesion depths according to DIFOTI show closer correlation with the reference standard than those recorded by film or digital radiography. These conclusions, drawn from an *in vitro* study, have certain limitations and should not be directly extrapolated to *in vivo* conditions. However, the promising results of the present study suggest that further investigation of the DIFOTI method under clinical conditions is warranted. 

## Figures and Tables

**Table 1:** Reproducibility of the three diagnostic methods, in form of intra-examiner agreement presented as mean *κ*_*w*_, and inter-examiner agreement presented as range of mean *κ*_*w*_ based on results from the eight observers.

Method	Intra-observer agreement	Inter-observer agreement
DIFOTI	0.82	0.62–0.76
Film radiography	0.82	0.63–0.78
Digital radiography	0.79	0.67–0.78

**Table 2:** Examiner performance in detecting approximal caries lesions at two different diagnostic thresholds, expressed as sensitivity, specificity and AUC: area under the receiver operating characteristic (ROC) curve. Confidence interval of 95% is presented in brackets (CI 95%).

Method of examination	Sensitivity		Specificity		AUC (CI 95%)	
	Cut off 1	Cut off 2	Cut off 1	Cut off 2	Cut off 1	Cut off 2
Examiner 1						
DIFOTI	0.65	0.33	0.91	0.95	0.79* (0.72–0.86)	0.80* (0.68–0.92)
Film	0.30	0.48	0.97	1.0	0.64* (0.58–0.70)	0.92* (0.82–1.00)
Digital	0.30	0.40	0.94	0.95	0.62* (0.54–0.69)	0.82* (0.69–0.94)
Examiner 2						
DIFOTI	0.83	0.73	0.85	0.90	0.87* (0.81–0.94)	0.90* (0.83–0.98)
Film	0.33	0.53	0.82	0.91	0.57 (0.48–0.66)	0.87* (0.77–0.97)
Digital	0.22	0.53	0.82	0.92	0.52 (0.44–0.61)	0.79* (0.65–0.92)
Examiner 3						
DIFOTI	0.62	0.60	0.94	0.94	0.79* (0.73–0.86)	0.86* (0.74–0.98)
Film	0.29	0.47	0.88	0.98	0.59* (0.51–0.66)	0.79* (0.65–0.93)
Digital	0.35	0.47	0.97	0.99	0.66* (0.60–0.73)	0.80* (0.66–0.94)
Examiner 4						
DIFOTI	0.46	0.33	1.0	1.0	0.73* (0.67–0.79)	0.89* (0.79–1.00)
Film	0.13	0.27	1.0	0.99	0.56* (0.52–0.60)	0.65* (0.52–0.78)
Digital	0.24	0.40	0.97	0.99	0.60* (0.54–0.66)	0.78* (0.64–0.92)
Examiner 5						
DIFOTI	0.89	0.40	0.68	0.95	0.83* (0.75–0.91)	0.89* (0.83–0.96)
Film	0.27	0.40	0.85	0.99	0.56 (0.48–0.64)	0.79* (0.66–0.93)
Digital	0.43	0.60	1.0	1.0	0.71* (0.65–0.78)	0.98* (0.95–1.00)
Examiner 6						
DIFOTI	0.89	0.93	0.65	0.87	0.88* (0.82–0.94)	0.95* (0.91–0.99)
Film	0.60	0.47	0.56	1.0	0.63* (0.53–0.73)	0.88* (0.77–0.98)
Digital	0.64	0.47	0.74	0.96	0.71* (0.61–0.80)	0.88* (0.77–0.98)
Examiner 7						
DIFOTI	0.71	0.60	0.91	0.82	0.82* (0.74–0.89)	0.83* (0.72–0.93)
Film	0.38	0.47	0.94	0.96	0.65* (0.58–0.73)	0.88* (0.78–0.99)
Digital	0.40	0.47	0.91	0.95	0.65* (0.57–0.73)	0.90* (0.82–0.99)
Examiner 8						
DIFOTI	0.62	0.67	1.0	0.99	0.81* (0.75–0.87)	0.91* (0.82–1.00)
Film	0.33	0.53	0.91	0.99	0.62* (0.55–0.70)	0.87* (0.75–0.99)
Digital	0.35	0.47	0.94	0.99	0.64* (0.57–0.72)	0.79* (0.65–0.94)

Cut-off 1: Diagnostic threshold D1; enamel and dentin caries lesions.

Cut-off 2: Diagnostic threshold D3; dentin caries lesions.

*Statistically significant *P* < 0.05.

**Table 3:** Comparison of methods by Wilcoxon matched pairs test. The table presents *P*-values.

		DIFOTI-film	DIFOTI-digital	Film-digital
Cut off 1	Sensitivity	0.0117	0.0117	0.1762
	Specificity	0.6726	0.6121	0.3525
	AUC	0.0117	0.0117	0.1508

Cut off 2	Sensitivity	0.1282	0.1834	0.4652
	Specificity	0.0346	0.0464	0.5294
	AUC	0.2626	0.2626	0.7353

**Table 4:** Agreement with reference standard. The table presents weighted kappa values.

Method of examination	Examiners							
	1	2	3	4	5	6	7	8
DIFOTI	0.56	0.75	0.68	0.57	0.65	0.75	0.60	0.73
Film	0.61	0.46	0.49	0.36	0.44	0.65	0.59	0.58
Digital	0.45	0.37	0.56	0.47	0.71	0.63	0.56	0.53
